# Chinese Poplar Propolis Inhibits MDA-MB-231 Cell Proliferation in an Inflammatory Microenvironment by Targeting Enzymes of the Glycolytic Pathway

**DOI:** 10.1155/2021/6641341

**Published:** 2021-02-15

**Authors:** Junya Li, Hui Liu, Xinying Liu, Shengyu Hao, Zihan Zhang, Hongzhuan Xuan

**Affiliations:** ^1^School of Life Science, Liaocheng University, Liaocheng 252059, China; ^2^Center of Bee Industry on Seed-Breeding and Popularization in Shandong Province, Jinan 250010, China; ^3^School of Physical Science and Information Technology, Liaocheng University, Liaocheng 252059, China

## Abstract

Propolis is rich in flavonoids and has excellent antitumor activity. However, little is known about the potential effects of propolis on glycolysis in tumor cells. Here, the antitumor effects of propolis against human breast cancer MDA-MB-231 cells in an inflammatory microenvironment stimulated with lipopolysaccharide (LPS) were investigated by assessing the key enzymes of glycolysis. Propolis treatment obviously inhibited MDA-MB-231 cell proliferation, migration and invasion, clone forming, and angiogenesis. Proinflammatory mediators, including tumor necrosis factor-alpha (TNF-*α*), interleukin (IL)-1*β*, and IL-6, as well as NLRP3 inflammasomes, were decreased following propolis treatment when compared with the LPS group. Moreover, propolis treatment significantly downregulated the levels of key enzymes of glycolysis–hexokinase 2 (HK2), phosphofructokinase (PFK), pyruvate kinase muscle isozyme M2 (PKM2), and lactate dehydrogenase A (LDHA) in MDA-MB-231 cells stimulated with LPS. After treatment with 2-deoxy-D-glucose (2-DG), an inhibitor of glycolysis, the inhibitory effect of propolis on migration was not significant when compared with the LPS group. In addition, propolis increased reactive oxygen species (ROS) levels and decreased mitochondrial membrane potential. Taken together, these results indicated that propolis targeted key enzymes of glycolysis to suppress the proliferation of MDA-MB-231 cells in an inflammatory microenvironment. These studies provide a molecular basis for propolis as a natural anticancer agent against breast cancer.

## 1. Introduction

Breast cancer (BC) is one of the most common malignant tumors and a major cause of cancer death among women worldwide, and the triple-negative breast cancer (TNBC) subtype is the most aggressive one [1]. Worldwide statistics showed that in 2018, approximately two million new cases were detected, with the total BC cases accounting for 11.6% of all cancers. Therefore, the search for effective anticancer agents is urgent for BC therapy and to improve the quality of life of patients.

In recent years, the antitumor activities of flavonoids have attracted increasing interest among researchers. Propolis, rich in flavonoids, is a resinous substance collected by honeybees (*Apis mellifera*) from various plant sources. It has been used as a folk medicine since ancient times [2].

According to its different plant sources, propolis can be divided into five categories: *Populus* propolis, *Baccharis* propolis*, Clusia* propolis, *Macaranga* propolis, and Mediterranean propolis [3]. More than 200 flavonoids have been identified from various kinds of propolis around the world [4]. Chinese propolis (CP), one of the *Populus* type of propolis, mainly contains flavonoids and phenolic compounds and has exhibited extensive pharmacological activities including antibacterial [5], anti-inflammatory [6], antivirus [7], antitumor [8], antioxidant [9], and immunoregulation activities [10].

We and other researchers demonstrated that propolis has an excellent antitumor activity against various tumor cell lines in vivo and in vitro [8, 11]. Furthermore, we reported that Chinese propolis and its major constituent—caffeic acid phenethyl ester (CAPE)—inhibit breast cancer cell proliferation in an inflammatory microenvironment by inhibiting the Toll-like receptor 4 (TLR4) signal pathway and inducing apoptosis and autophagy [12]. However, these antitumor mechanisms have still not been fully elucidated.

The mitochondria are the center of energy and metabolism in eukaryons. Warburg revealed the unique energy metabolism in cancer cells, suggesting a shift in energy production from mitochondrial oxidative phosphorylation (OXPHOS) to aerobic glycolysis [13]. Alterations in the glucose metabolism are characterized by increased uptake of glucose, hyperactivated glycolysis, decreased OXPHOS component, and the accumulation of lactate. Cancer cells rely on higher rates of aerobic glycolysis as their primary source of energy; thus, aerobic glycolysis becomes a hallmark of cancer cells. Key enzymes of glycolysis, namely, hexokinase 2 (HK2), phosphofructokinase (PFK), pyruvate kinase muscle isozyme M2 (PKM2), and lactate dehydrogenase A (LDHA), are critical glycolysis regulators [14]. Enhanced glycolysis correlates with the upregulation and activation of critical glycolytic enzymes, which, in turn, promotes proliferation, metastasis, and tumorigenesis [15]. Inhibition of glycolysis has been identified as a novel therapeutic focus in cancer therapies.

The levels of HK2, PFK, PKM2, and LDHA have been individually reported to be correlated with cancer cell growth [16–19]. Propolis has excellent antitumor activities, and whether propolis could target crucial glycolytic enzymes to inhibit tumor cell proliferation is still unclear. In the present study, the roles of Chinese propolis on key glycolytic enzymes—HK2, PFK, PKM2, and LDHA—were assessed in MDA-MB-231 cells stimulated with lipopolysaccharide (LPS).

## 2. Materials and Methods

### 2.1. Chemicals and Reagents

Leibovitz's L15 medium and fetal bovine serum (FBS) were purchased from Gibco-BRL (USA). LPS from *Escherichia coli* 055 : B5,2′,7′-dichlorodihydrofluorescein diacetate (DCFH-DA) and JC-1 was obtained from Sigma-Aldrich (St. Louis, USA). Matrigel basement membrane matrix was obtained from BD Biocoat (USA). The Enhanced Cell Counting Kit-8 was obtained from Beyotime (China). Primary antibodies against *β*-actin, GAPDH, HK2, PFK, PKM2, LDHA, NLRP3, and secondary antibody were obtained from ABclonal Biotech (USA). Enzyme-linked immunosorbent assay (ELISA) kits for HK2, PFK, PKM2, and LDHA were obtained Shanghai Enzyme-linked Biotechnology Co., Ltd. (China). A secondary antibody for immunofluorescence and donkey anti-rabbit IgG Alexa Fluor-488 was purchased from Life Technologies (USA).

### 2.2. Preparation of Chinese Propolis Extract

Chinese propolis was collected from Nanyang in Henan province, located in North China in 2017 (voucher specimen no. CP17110702), and the main plant origin of the propolis sample collected was poplar (*Populus* sp.). The propolis was firstly frozen and then extracted with 95% (v/v) ethanol. The extracted propolis was then ultrasonicated at 40°C for 3 h. The supernatant of the extracted propolis was filtered with filter papers to remove the residues, and then the propolis was extracted again three times. Thereafter, all of the supernatants were combined and evaporated with a rotary evaporator under reduced pressure at 50°C. Then, the concentrate was further evaporated in an oven at 50°C until reaching a constant weight and was stored at -20°C. The ethanol extracted Chinese propolis (EECP) was redissolved in ethanol before use. The major chemical constituents of the EECP were analyzed via HPLC-DAD/Q-TOF-MS as previously described [20].

### 2.3. Cell Culture

The human breast cancer cell line MDA-MB-231 was purchased from Cell Bank of Typical Culture Preservation Committee, Chinese Academy of Sciences, Shanghai (Shanghai, China). Cells were cultured in Leibovitz's L15 medium supplemented with 10% (v/v) FBS, 100 U/mL of penicillin, and 100 *μ*g/mL streptomycin at 37°C.

### 2.4. Exposure of MDA-MB-231 to EECP

When the MDA-MB-231 cells reached 80%—90% confluence, they were divided into 3 groups for treatment: (a) culture in L15 medium (control group), (b) culture in L15 medium with 1 *μ*g/mL LPS (LPS group), and (c) culture in L15 medium with 1 *μ*g/mL LPS and EECP (25, 50, and 100 *μ*g/mL) (test group). EECP was dissolved in ethanol and applied to the cells, with a final ethanol concentration in the culture medium of <0.1% (v/v). Ethanol at a concentration of 0.1% (v/v) did not affect the cell viability.

### 2.5. Cell Viability Assay

Cell viability was measured using the CCK-8 kit. Cells (1 × 10^5^ cells/well) were seeded in 96-well plates. When cells reached 60-70% confluence, they were treated with or without EECP (25, 50, and 100 *μ*g/mL) and LPS (1 *μ*g/mL). At 12, 24, and 48 h, cell viabilities were measured following the manufacturer's instructions. The optical density was determined at 450 nm.

### 2.6. Transwell Analysis

MDA-MB-231 cells were seeded into 6-wells plates. The medium was replaced with fresh complete medium or medium containing 1 *μ*g/mL LPS alone or LPS with EECP (25, 50, and 100 *μ*g/mL) when cells reached 70% confluence. The cells were further incubated for 24 h. Thereafter, the cells were treated with trypsin, resuspended in serum-free medium, and seeded into the upper chamber of the Transwell. Serum-free medium containing 5 × 10^4^ cells was added to the upper chamber for migration assays, whereas 1 × 10^5^ cells were used for Matrigel invasion assays. L15 medium with 20% FBS was added to the lower chamber. After incubation for 24 h, the cells were fixed with 95% (v/v) ethanol for 10 min, then stained with 0.1% crystal violet solution for 30 min and pictured under a microscope. The migration and invasion rates of cells were counted using Image J software.

### 2.7. Endothelial Cell Tube Formation Assay

Matrigel was diluted with serum-free medium, and 200 *μ*L of diluent was added into a 24-well plate and maintained at 37°C for 1 hour. Then, 1 × 10^5^ human umbilical vein endothelial cells (HUVECs) were treated with trypsin, resuspended in serum-free medium, and gently seeded on Matrigel-coated wells. Two hours later, cells were treated with EECP (25, 50, or 100 *μ*g/mL) and LPS (1 *μ*g/mL). Endothelial cell tube formation was photographed through an inverted microscope at 6 h. The total tube numbers and branches were calculated using ImageJ software.

### 2.8. Colony Formation Assay

MDA-MB-231 cells were seeded into a 6-well plate at 1 × 10^3^ cells/well and cultured for 24 h. Then, cells were treated with EECP (25, 50, and 100 *μ*g/mL) and LPS (1 *μ*g/mL) for 24 h. After that, the medium was replaced with fresh complete medium. The medium was changed every three days. Ten days later, cell colonies were washed with phosphate-buffered saline and fixed with 95% (v/v) ethanol. Then, cells were stained with 0.1% crystal violet and captured under an inverted optical microscope. ImageJ software was used to count the numbers of cells.

### 2.9. Western Blot Assay

After treatment with EECP (25, 50, and 100 *μ*g/mL) for 24 h, the total protein was extracted using a commercial protein extraction kit. The protein concentration was measured using a BCA protein assay kit. Subsequently, equal amounts of protein (30 *μ*g) were separated via 12% SDS-PAGE. The gels were then transferred to polyvinylidene fluoride (PVDF) membranes. Skim milk (5%) was used to block the nonspecific binding sites for 1 h at room temperature. Primary antibodies (HK2, PFK, PKM2, LDHA, and NLRP3) were incubated with the membranes at 4°C overnight, and horseradish peroxidase- (HRP-) conjugated secondary antibodies were then applied for another 1 h of incubation at room temperature. The immunoreactive signals were detected under an Amersham Image 600 (USA), and the relative quantity of protein was analyzed using ImageJ software.

### 2.10. Reverse Transcription-Quantitative Polymerase Chain Reaction (RT-PCR) Assay

After treatment with EECP (25, 50, and 100 *μ*g/mL) for 24 h, the total RNA of cells was extracted using an RNA extraction kit (Carry Helix, China) according to the manufacturer's protocol. Then, the cDNA was reversed from RNA using a PrimeScript RT Kit (Thermo #K1622). The primers used in the present study are listed in Table 1. Quantitative real-time PCR was performed using the SYBR Green PCR reagent Kit (Thermo F-415XL). The expression of the housekeeping gene GAPDH was used to normalize the expression levels, and the results were expressed as 2^-*ΔΔ*Ct^.

### 2.11. ELISA Assay

The levels of glycolytic key enzymes-HK2, PFK, PKM2, and LDHA and proinflammatory cytokines-TNF-*α*, IL-1*β*, and IL-6 in cell supernatant after EECP (25, 50, and 100 *μ*g/mL) treatment were measured using commercial ELISA kits following standard protocols.

### 2.12. Immunofluorescence Assay

After treatment with EECP (25, 50, and 100 *μ*g/mL) for 24 h, cells were fixed with 4% paraformaldehyde (w/v) at room temperature for 15 min, then blocked with 5% donkey serum (v/v) for 20 min. After adding the primary antibodies for PKM2 and LDHA (1 : 100) and secondary antibody (1 : 200) (FITC-IgG), a laser scanning confocal microscope (Olympus FV1200, Japan) was used for fluorescence detection. For analysis, ImageJ was used as software. Images are representative of three independent experiments.

### 2.13. Reactive Oxygen Species (ROS) and Mitochondrial Membrane Potential Assay

The fluorescent probes, DCFH-DA and JC-1, were used to test ROS production and mitochondrial membrane potential, respectively, according to the manufacturer's protocol. The levels of ROS and mitochondrial membrane potential were quantified using the software accompanying laser scanning confocal microscope (Olympus FV1200, Japan). ROS results were shown as the relative fluorescence intensity ratio compared with the LPS group, and mitochondrial membrane potential results were shown as the ratio of red to green fluorescence as compared with the LPS group.

### 2.14. Statistical Analysis

All experiments were repeated at least three times independently. Data were expressed as the mean ± SEM. Statistical analysis involved paired Student's *t*-test and ANOVA via SPSS version 18.0 and Graphpad Prism 5. A *P* value of <0.05 was considered to indicate a statistically significant difference.

## 3. Results

### 3.1. The Major Chemical Components of EECP

The chemical constituents of EECP were measured by HPLC-DAD/Q-TOF-MS analysis, and a total of 16 constituents were identified and quantified (Table 2). Flavonoids such as chrysin, pinocembrin, pinobanksin, apigenin, galangin, and quercin were rich in EECP, and previous studies also showed that these compounds have excellent antitumor activities [21–25].

### 3.2. EECP Decreased Cell Viability in MDA-MB-231 Cells Stimulated with LPS

**Figure 1:**
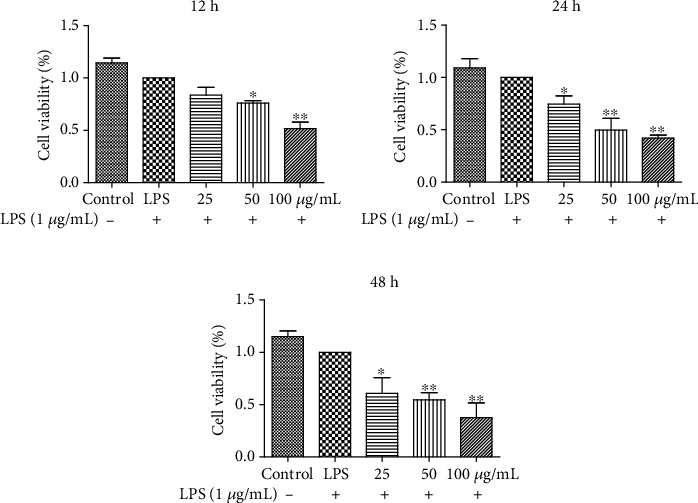
EECP decreased cell viability in MDA-MB-231 cells stimulated with lipopolysaccharide (LPS). (a)–(c) Effect of EECP on the cell viability of MDA-MB-231 cells stimulated with LPS at 12, 24, and 48 h, respectively. 25, 50, and 100 *μ*g/mL: cells treated with EECP at 25, 50, and 100 *μ*g/mL, respectively. Values represent the mean ± SEM from three independent experiments (^∗^*P* < 0.05, ^∗∗^*P* < 0.01  vs. control, *n* = 3).

To investigate the antiproliferation activity of EECP (25, 50, and 100 *μ*g/mL) in MDA-MB-231 cells stimulated with LPS, the cell viabilities at 12, 24, and 48 h were firstly tested using a CCK-8 kit. As shown in Figure 1, there was dramatic decrease in cell viabilities after treatment with different concentrations of EECP, and EECP was found to inhibit MDA-MB-231 cell proliferation in a time- and dose-dependent manner when compared with the LPS group. There was no significant different in cell viabilities between the control and LPS groups (^∗^*P* < 0.05, ^∗∗^*P* < 0.01; Figures 1(a)–1(c)).

### 3.3. EECP Suppressed Migration, Invasion, and Colony Formation in MDA-MB-231 Cells Stimulated with LPS

**Figure 2:**
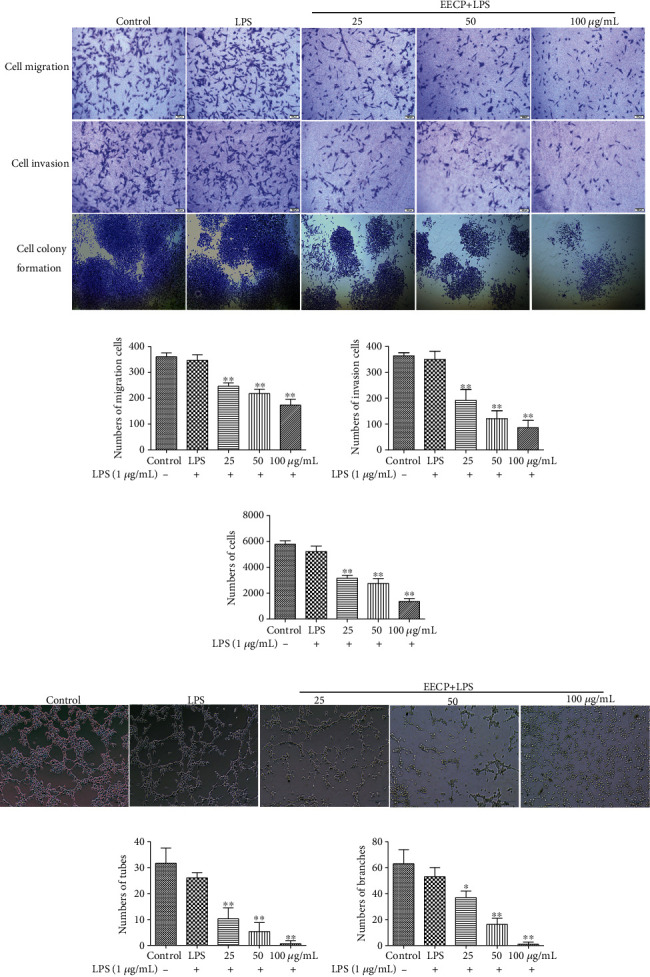
EECP suppressed migration, invasion, and colony formation in MDA-MB-231 cells stimulated with LPS. (a) EECP suppressed migration, invasion, and colony formation in MDA-MB-231 cells. (b)–(d) Quantification of cell migration, invasion, and colony formation in MDA-MB-231 cells after EECP treatment. (e) EECP inhibited angiogenesis in human umbilical vein endothelial cells (HUVECs) at 6 h. (f, g) Quantification of tubes and branches of angiogenesis. Values represent the mean ± SEM from three independent experiments (^∗^*P* < 0.05, ^∗∗^*P* < 0.01 vs. control, *n* = 3).

To further confirm the effect of EECP on the migration, invasion, and clone formation of MDA-MB-231 cells stimulated with LPS, a transwell experiment and angiogenesis assay were performed. In comparison with the control and LPS groups, treatment with different concentrations of EECP obviously suppressed the cell migration and invasiveness of MDA-MB-231 cells stimulated with LPS. Pretreatment with different concentrations of EECP also dramatically decreased the numbers of colonies formed compared with the LPS group (^∗^*P* < 0.05, ^∗∗^*P* < 0.01; Figures 2(a)–(d)).

### 3.4. EECP Inhibited Endothelial Cell Tube Formation

Tumor associated angiogenesis plays a crucial role in the growth and metastasis of tumor [26]. To determine the effect of EECP on angiogenesis in vitro, HUVECs were treated with different concentrations of EECP (25, 50, and 100 *μ*g/mL) for 6 h. Compared with the LPS group, endothelial cell tube formation abilities were significantly decreased after treatment with EECP. Correspondingly, the numbers of tubes and tube branches were significantly decreased in a dose-dependent manner after EECP treatment, suggesting that EECP probably inhibits tumor angiogenesis (^∗^*P* < 0.05, ^∗∗^*P* < 0.01; Figures 2(e)–(g)).

### 3.5. EECP Suppressed the Levels of Inflammatory Mediators in MDA-MB-231 Cells Stimulated with LPS

**Figure 3:**
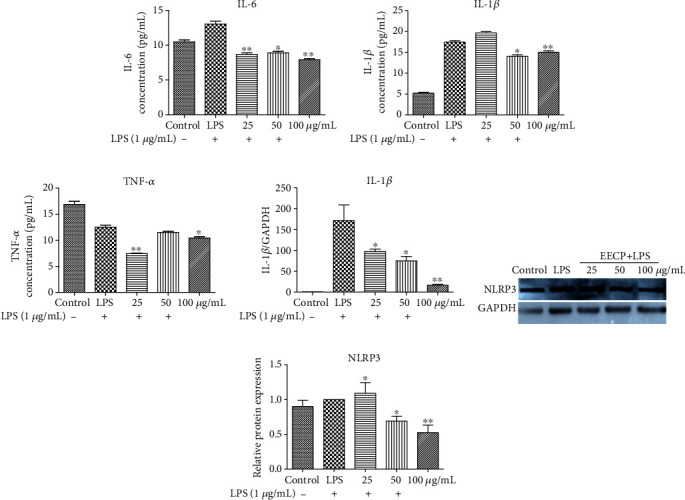
EECP suppressed the levels of inflammatory mediators in MDA-MB-231 cells stimulated with LPS. (a)–(c) EECP inhibited the levels of IL-6, IL-1*β*, and TNF-*α* in MDA-MB-231 cells stimulated with LPS, as detected by ELISA kits. (d) EECP inhibited the levels of IL-1*β* compared with the LPS group, as detected by RT-PCR. (e) EECP inhibited the levels of NLRP3 compared with the LPS group, as detected by Western blotting. (f) Quantification of the relative expression level of NLRP3 in MDA-MB-231 cells. Values represent the mean ± SEM from three independent experiments (^∗^*P* < 0.05, ^∗∗^*P* < 0.01 vs. control, *n* = 3).

The levels of IL-6, IL-1*β*, and TNF-*α* in the LPS groups were evidently increased. Treatment with EECP resulted in a decrease in these proinflammatory cytokines when compared with the LPS group (Figures 3(a)–3(c)). Furthermore, a decrease in the production of IL-1*β* was also demonstrated by RT-PCR (Figure 3(d)). More importantly, treatment with a higher concentration of EECP significantly reversed the increase of NLRP3, as shown by Western blotting analysis (Figures 3(e) and 3(f)).

### 3.6. EECP Decreased the Levels of HK2, PFK, PKM2, and LDHA in MDA-MB-231 Cells Stimulated with LPS

**Figure 4:**
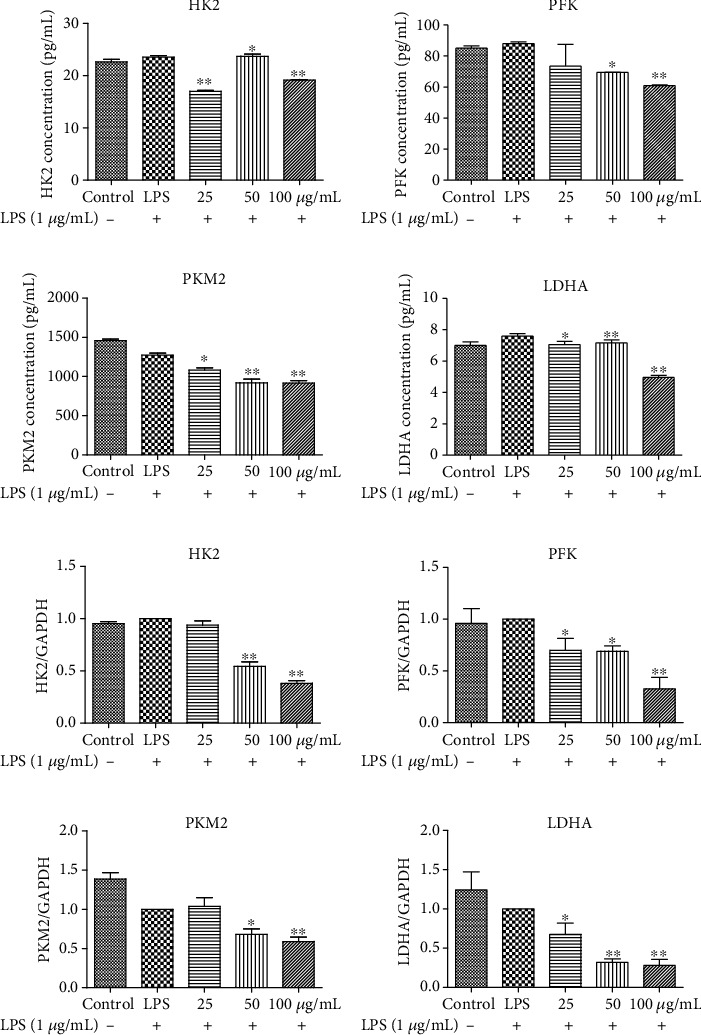
EECP decreased the levels of HK2, PFK, PKM2, and LDHA in MDA-MB-231 cells stimulated with LPS. (a)–(d) EECP decreased the levels of HK2, PFK, PKM2, and LDHA in MDA-MB-231 cells, as detected by ELISA kits. (e)–(h) EECP decreased the levels of HK2, PFK, PKM2, and LDHA in MDA-MB-231 cells, as detected by RT-PCR. Values represent the mean ± SEM from three independent experiments (^∗^*P* < 0.05, ^∗∗^*P* < 0.01 vs. control, *n* = 3).

The levels of the key enzymes of glycolysis—HK2, PFK, PKM2, and LDHA—in MDA-MB-231 cells stimulated with LPS were firstly measured using ELISA kits. The levels of HK2, PFK, PKM2, and LDHA after EECP treatment were obviously decreased compared with the LPS group, especially after treatment with a higher concentration of EECP (Figures 4(a)–4(d)).

The decrease in the levels of HK2, PFK, PKM2, and LDHA in MDA-MB-231 cells stimulated with LPS was further confirmed by RT-PCR (Figures 4(e)–4(h)). As expected, EECP treatment dramatically decreased the mRNA levels of HK2, PFK, PKM2, and LDHA in a dose-dependent manner compared with the LPS group.

**Figure 5:**
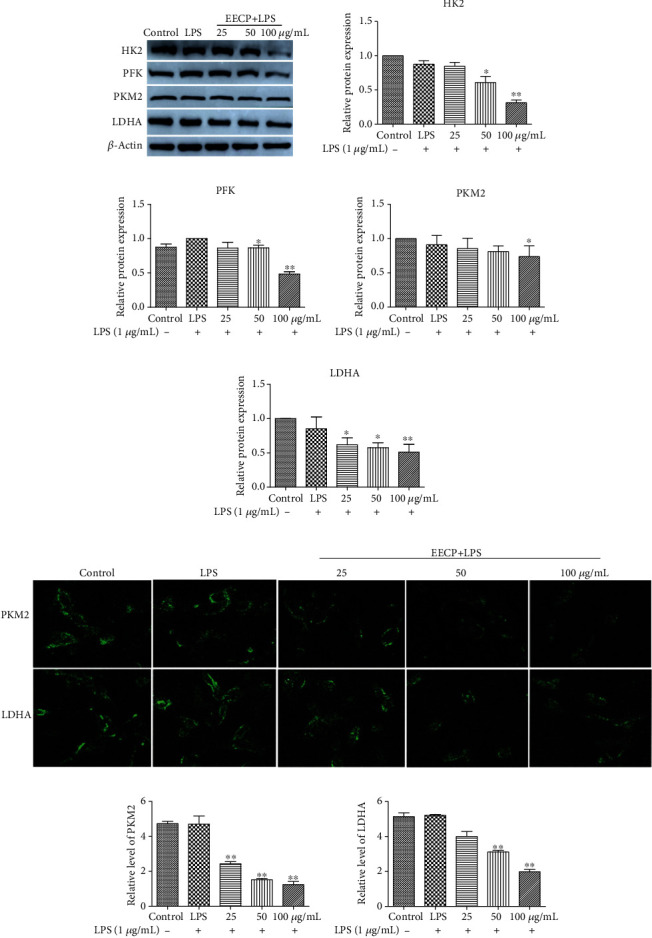
EECP decreased the levels of HK2, PFK, PKM2, and LDHA in MDA-MB-231 cells stimulated with LPS, as detected by Western blotting and immunofluorescence assay. (a) The protein expression levels of HK2, PFK, PKM2, and LDHA in MDA-MB-231 cells. (b)–(e) Quantification of the relative expression levels of HK2, PFK, PKM2, and LDHA in MDA-MB-231 cells. (f) Expression levels of PKM2 and LDHA, as detected by the immunofluorescence assay. (g, h) Quantification of the relative expression levels of PKM2 and LDHA in MDA-MB-231 cells. Values represent the mean ± SEM from three independent experiments (^∗^*P* < 0.05, ^∗∗^*P* < 0.01 vs. control, *n* = 3).

The protein expression levels of HK2, PFK, PKM2, and LDHA in MDA-MB-231 cells stimulated with LPS were also assessed. As shown in Figures 5(a)–5(e), it was observed that expression levels of the key enzymes of glycolysis were downregulated in the EECP treatment groups compared with the LPS group. The EECP administration alleviated glycolysis by suppressing the levels of HK2, PFK, PKM2, and LDHA.

The immunofluorescence assay of PKM2 and LDHA further confirmed that the EECP administration suppressed the protein expression of PKM2 and LDHA in MDA-MB-231 cells stimulated with LPS. The fluorescence intensities of PKM2 and LDHA evidently decreased in EECP treatment groups compared with the LPS group (^∗^*P* < 0.05, ^∗∗^*P* < 0.01; Figures 5(f)–5(h)).

### 3.7. EECP Increased ROS Production and Decreased Mitochondrial Membrane Potential in MDA-MB-231 Cells Stimulated with LPS

**Figure 6:**
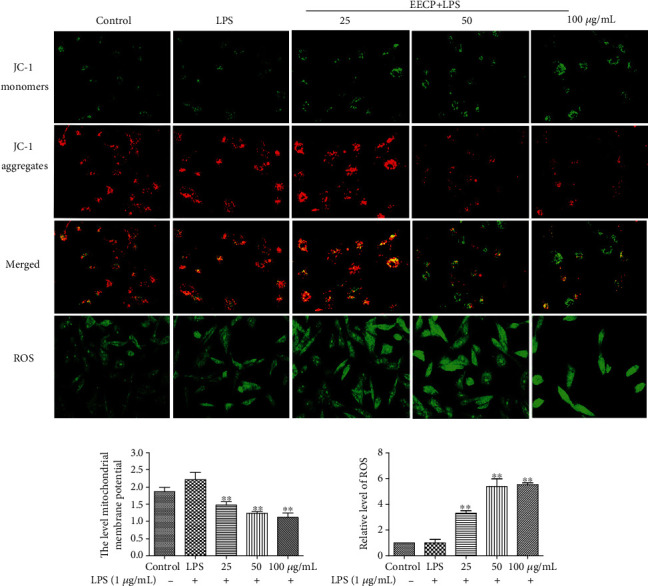
EECP increased reactive oxygen species (ROS) levels and decreased mitochondrial membrane potential in MDA-MB-231 cells stimulated with LPS. (A) Fluorescence micrographs obtained at 24 h. (b, c) Values represent the relative fluorescence intensity per cell determined by laser scanning confocal microscopy. Values represent the mean ± SEM from three independent experiments (^∗^*P* < 0.05^∗∗^*P* < 0.01, vs. control, *n* = 3).

Mitochondria are the center of energy and metabolism in eukaryons. They are also cellular organs with a critical adjusting function in apoptosis signaling processes. To determine the effect of EECP on mitochondria, the mitochondrial membrane potential and ROS production were analyzed. The functions of mitochondria were damaged, and ROS production was obviously increased after EECP treatment with the LPS group in MDA-MB-231 cells stimulated with LPS (^∗^*P* < 0.05, ^∗∗^*P* < 0.01; Figures 6(a)–6(c)).

### 3.8. EECP Inhibited MDA-MB-231 Cell Migration in a Glycolysis—Dependent Manner

**Figure 7:**
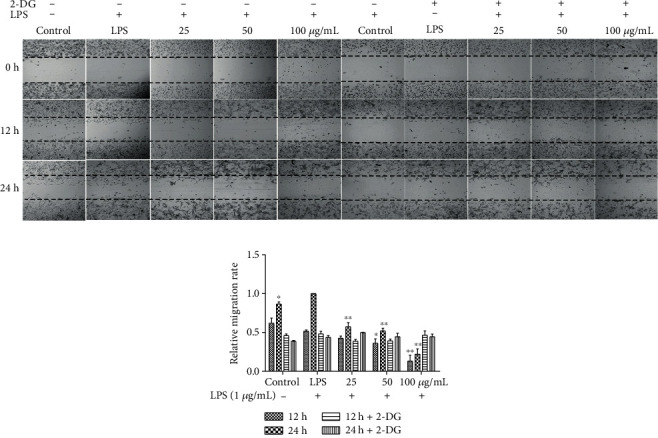
EECP inhibited MDA-MB-231 cell migration in a glycolysis-dependent manner. (a) Effect of EECP on the migration of MDA-MB-231 cells stimulated with LPS with or without 2-deoxy-D-glucose (2-DG). (b) Quantification of the relative migration rate of MDA-MB-231 cells. Values represent the mean ± SEM from three independent experiments (^∗^*P* < 0.05, ^∗∗^*P* < 0.01 vs. control, *n* = 3).

To confirm whether EECP inhibited MDA-MB-231 cell migration through inhibiting glycolysis, the scratching assay was performed by adding 2-deoxy-D-glucose (2-DG) into cells. EECP significantly suppressed MDA-MB-231 cell migration without 2-DG in the medium. However, the inhibitory effects in EECP groups were not significant compared with the LPS group after adding 2-DG into the medium, suggesting that EECP's exhibitory effects on MDA-MB-231 cells were probably via the glycolysis pathway (^∗^*P* < 0.05, ^∗∗^*P* < 0.01; Figures 7(a) and 7(b)).

## 4. Discussion

Although propolis has been shown to have excellent antitumor activities, previous studies assessing antitumor mechanisms by targeting key enzymes of glycolysis were limited. In the present study, we evaluated the antitumor mechanisms of Chinese *populus* propolis in MDA-MB-231 cells stimulated with LPS by studying key glycolysis-related enzymes: HK2, PFK, PKM2, and LDHA. Propolis treatment was able to inhibit MDA-MB-231 cells proliferation, migration, invasion, and angiogenesis, as well as suppress the production of proinflammatory cytokines. More importantly, propolis treatment obviously inhibited the levels of HK2, PFK, PKM2, and LDHA.

The incidence of BC has grown rapidly in recent years. BC can severely decrease a patient's quality of life and has high lethality in women. Tumor glycolysis is crucial for the efficient management of cellular bioenergetics and uninterrupted cancer growth [27]. Although glycolysis is less efficient than oxidative phosphorylation in terms of the net yield of ATP, cancer cells adapt to this mathematical disadvantage by increased glucose uptake, which in turn facilitates a higher rate of glycolysis. Glycolysis in tumor cells produces pyruvate, which is converted to lactic acid in the cytoplasm. These acidic products alter the microenvironment to accelerate tumor proliferation, migration, invasion, and angiogenesis and instigate immunosuppressive networks that are pivotal for cancer cells to escapes immune surveillance [28]. Multiple lines of evidence have established that higher expression levels of key enzymes such as HK2, PFK, PKM2, and LDHA are linked to malignant growth [29, 30]. Thus, targeting glycolysis remains an attractive strategy for therapeutic intervention.

HK catalyzes the first step in glucose metabolism converting glucose to glucose-6-phosphate [29]. HK2 status is clinically linked to recurrence and poor prognosis in BC [31]. This enzyme plays a pivotal role in tumor glycolysis. Inhibition of HK2 has been shown to inhibit the proliferation of cancer cells by shifting the glycolytic pathway with reduced lactate formation [32]. Here, the levels of HK2 were significantly decreased in MDA-MB-231 cells after propolis treatment, as determined by multiple measurement methods.

PFK is crucial in BC cancer progression and is also upregulated in cancer cells; it catalyzes another rate-limiting step of glycolysis, from fructose-6-phosphate to fructose-1,6-bisphosphate [33]. Consistent with the results for HK2, propolis treatment obviously suppressed the levels of PFK, suggesting that propolis could target key glycolytic enzymes.

PK catalyzes the conversion of phosphoenolpyruvate (PEP) to pyruvate. Among its various isoforms, the M2 isoform has gained much attention due to its higher expression in tumor cells [34]. PKM2 is crucial for aerobic glycolysis and tumor energy metabolism [35]. Emerging preclinical studies have indicated that PKM2 could represent a potential therapeutic target [36]. Here, we also demonstrated that propolis treatment alleviated the levels of PKM2.

LDH catalyzes the final step in the glycolytic pathway that converts pyruvate into lactate, and a higher lactate level significantly correlates with tumor recurrence and the metastatic potential of tumors resulting in poor patient outcomes [37]. Besides this, several studies have identified a prominent role of LDHA in TNBCs [38]. Propolis significantly inhibited the levels of LDHA, demonstrating promising effects in the prevention of TNBCs.

2-DG, a glucose analog, inhibits glycolysis via its actions on hexokinase [39]. We used 2-DG to inhibit glycolysis in MDA-MB-231 cells to assess the effects of propolis. The results showed that propolis treatment no longer inhibited MDA-MB-231 cell migration compared with the LPS group after inhibition of glycolysis, indicating that propolis exerts the antitumor activity by targeting glycolysis.

ROS are a key determinant of cancer's metabolic phenotype [40]. Maintaining ROS within a narrow range allows malignant cancer cells to enhance their growth and invasion while limiting their apoptotic susceptibility [41]. Cancer cells actively modify their metabolism to optimize intracellular ROS levels and thereby improve survival [42]. Propolis treatment evidently increased the ROS level and decreased the mitochondrial membrane potential in MDA-MB-231 cells stimulated with LPS. The present results are consistent with our original findings, indicating that propolis increases the ROS level to promote cancer cells apoptosis.

The inhibition of glycolysis can also transform tumor cells into forms that are sensitive to immunotherapy and can alter the tumor microenvironment [43]. The NLRP3 inflammasome can be activated by various danger-associated molecular patterns, and the activation of NLPR3 induces caspase-1 activation, IL-1*β* or IL-18 secretion, and pyroptosis [44]. It has been shown that NLRP3 inflammasome activation in the tumor microenvironment has a critical role in the response to some chemotherapeutic agents [45]. Propolis has excellent anti-inflammatory and immune regulation activities [10]. Here, we also demonstrated that propolis decreased the levels of proinflammatory mediators, including TNF-*α*, IL-1*β*, and IL-6, as well as NLRP3 inflammasome to improve the tumor inflammatory microenvironment.

Propolis is rich in flavonoids such as chrysin, pinocembrin, pinobanksin, apigenin, galangin, and quercin, and previous studies also showed that these compounds have excellent antitumor activities. Besides these, CAPE, one of the most important constituent of propolis, also has a strong antitumor activity. In all, these antitumor constituents in propolis account for the strong antitumor activities of propolis.

There are still some limitations in our study. First, although propolis has significant inhibitory effects on glycolytic key enzymes in MDA-MB-231 cells, the effects of propolis on these key enzymes in other tumor cell lines should be further demonstrated. Second, whether propolis attenuating glycolytic key enzymes suppresses the tumor growth signaling pathway such as PI3K-Akt or propolis inhibiting the PI3K-Akt signaling pathway attenuates glycolytic key enzymes should be further studied.

## 5. Conclusions

Taken together, the results of this study show that propolis treatment of MDA-MB-231 cells in an inflammatory microenvironment was able to inhibit tumor cell proliferation by targeting key enzymes of glycolysis. As a natural product rich in flavonoids, propolis demonstrated good anti-inflammatory activity in the tumor microenvironment by inhibiting inflammatory cytokines. It was also noted that propolis could target key enzymes of glycolysis to suppress proliferation, migration, invasion, and angiogenesis. Moreover, it was demonstrated that propolis could damage the mitochondrial function by decreasing the mitochondrial membrane potential and increasing ROS production. As a result, Chinese *populus* propolis has excellent potential for use in the prevention and treatment of BC.

## Figures and Tables

**Table 1:** Primer sequences for genes.

Gene (R)	Sequence
LDHA	F: 5′-TTCAGCCCGATTCCGTTAC-3′
	R: 5′-AGACACCAGCAACATTCATTCC-3′

HK2	F: 5′-GCTTGCCTACTTCTTCACG-3′
	R:5′-TTTCTCCATCTCCTTGCG-3′

PFK	F:5′-ACAGAAGCCTTGGTCTAACAC-3′
	R:5′-GGAGAGTTGGAGGAATCAGTAG-3′

PKM2	F:5′-CCAGGTGAAGCAGAAAGGT-3′
	R:5′-CGGATGAATGACGCAAACA-3′

GAPDH	F:5′-AGAAGGCTGGGGCTCATTTG-3′
	R:5′-AGGGGCCATCCACAGTCTTC-3′

IL-1*β*	F: 5′-GCTCGCCAGTGAAATGATG-3′
	R: 5′-TGGTGGTCGGAGATTCGTAG-3′

**Table 2:** The chemical constituents of EECP identified by HPLC-DAD/Q-TOF-MS analysis.

Compounds	M + H	RT	Content (mg/g)
Chrysin	255.0652	31.264	16.887
Pinocembrin	257.0808	30.431	15.243
Pinobanksin	273.0757	27.11	6.879
Apigenin	271.0601	28.781	4.552
Galangin	271.0601	31.521	23.538
Kaempferol	287.0550	28.418	3.321
Quercin	303.0499	26.811	1.229
Caffeic acid	181.0495	17.172	10.857
Gallic acid	171.0288	26.392	0.470
p-Coumaric acid	165.0546	20.232	4.369
3-O-Acetyl pinobanksin	315.0863	30.708	15.570
Naringin	273.0612	27.11	6.876
Ferulic acid	195.0652	21.391	1.567
3,4-Dimethoxycinnamic acid	209.0808	24.676	9.945
*Trans*-cinnamic acid	195.0652	21.391	6.222
Caffeic acid phenethyl ester	285.1121	31.273	2.851

## Data Availability

The data used to support the findings of this study are included within the article.

## Abbreviations

BC: Breast cancer
EECP: Ethanol extract of Chinese propolis
FBS: Fetal bovine serum
HK2: Hexokinase 2
HUVECs: Human umbilical vein endothelial cells
IL-1*β*: Interleukin-1*β*
LDHA: Lactate dehydrogenase A
LPS: Lipopolysaccharide
PFK: Phosphate fructose kinase
PKM2: Pyruvate kinase muscle isozyme M2
PVDF: Polyvinylidene difluoride
ROS: Reactive oxygen species
SDS-PAGE: Sodium dodecyl sulfate polyacrylamide gel electrophoresis
TNBC: Triple-negative breast cancer
TNF-*α*: Tumor necrosis factor-alpha.
